# Genome-wide interrogation of structural variation reveals novel African-specific prostate cancer oncogenic drivers

**DOI:** 10.1186/s13073-022-01096-w

**Published:** 2022-08-31

**Authors:** Tingting Gong, Weerachai Jaratlerdsiri, Jue Jiang, Cali Willet, Tracy Chew, Sean M. Patrick, Ruth J. Lyons, Anne-Maree Haynes, Gabriela Pasqualim, Ilma Simoni Brum, Phillip D. Stricker, Shingai B. A. Mutambirwa, Rosemarie Sadsad, Anthony T. Papenfuss, Riana M. S. Bornman, Eva K. F. Chan, Vanessa M. Hayes

**Affiliations:** 1 Ancestry and Health Genomics Laboratory, Charles Perkins Centre, School of Medical Sciences, Faculty of Medicine and Health, University of Sydney, Camperdown, NSW Australia; 2 Genomics and Epigenetics Theme, Garvan Institute of Medical Research, Darlinghurst, NSW Australia; 3 Human Phenome Institute, Fudan University, Shanghai, China; 4 Sydney Informatics Hub, University of Sydney, Sydney, NSW Australia; 5 School of Health Systems and Public Health, University of Pretoria, Pretoria, South Africa; 6 Endocrine and Tumor Molecular Biology Laboratory, Instituto de Ciências Básicas da Saúde, Universidade Federal do Rio Grande do Sul, Porto Alegre, Brazil; 7 Laboratory of Genetics, Instituto de Ciências Biológicas, Universidade Federal do Rio Grande, Rio Grande, Brazil; 8 Department of Urology, St. Vincent’s Hospital, Darlinghurst, NSW Australia; 9 Department of Urology, Sefako Makgatho Health Science University, Dr George Mukhari Academic Hospital, Medunsa, Ga-Rankuwa, South Africa; 10 Bioinformatics Division, The Walter and Eliza Hall Institute of Medical Research, Parkville, Victoria Australia; 11 Department of Medical Biology, University of Melbourne, Melbourne, Victoria Australia; 12 NSW Health Pathology, Sydney, Australia; 13 Faculty of Health Sciences, University of Limpopo, Turfloop Campus, Mankweng, South Africa

**Keywords:** Chromosomal instability, Prostate cancer, African ancestry, Advanced disease, Ethnic disparity, Whole genome sequencing

## Abstract

**Background:**

African ancestry is a significant risk factor for advanced prostate cancer (PCa). Mortality rates in sub-Saharan Africa are 2.5-fold greater than global averages. However, the region has largely been excluded from the benefits of whole genome interrogation studies. Additionally, while structural variation (SV) is highly prevalent, PCa genomic studies are still biased towards small variant interrogation.

**Methods:**

Using whole genome sequencing and best practice workflows, we performed a comprehensive analysis of SVs for 180 (predominantly Gleason score ≥ 8) prostate tumours derived from 115 African, 61 European and four ancestrally admixed patients. We investigated the landscape and relationship of somatic SVs in driving ethnic disparity (African *versus* European), with a focus on African men from southern Africa.

**Results:**

Duplication events showed the greatest ethnic disparity, with a 1.6- (relative frequency) to 2.5-fold (count) increase in African-derived tumours. Furthermore, we found duplication events to be associated with *CDK12* inactivation and *MYC* copy number gain, and deletion events associated with *SPOP* mutation. Overall, African-derived tumours were 2-fold more likely to present with a hyper-SV subtype. In addition to hyper-duplication and deletion subtypes, we describe a new hyper-translocation subtype. While we confirm a lower *TMPRSS2-ERG* fusion-positive rate in tumours from African cases (10% *versus* 33%), novel African-specific PCa ETS family member and *TMPRSS2* fusion partners were identified, including *LINC01525, FBXO7*, *GTF3C2*, *NTNG1* and *YPEL5*. Notably, we found 74 somatic SV hotspots impacting 18 new candidate driver genes, with *CADM2*, *LSAMP*, *PTPRD*, *PDE4D* and *PACRG* having therapeutic implications for African patients.

**Conclusions:**

In this first African-inclusive SV study for high-risk PCa, we demonstrate the power of SV interrogation for the identification of novel subtypes, oncogenic drivers and therapeutic targets. Identifying a novel spectrum of SVs in tumours derived from African patients provides a mechanism that may contribute, at least in part, to the observed ethnic disparity in advanced PCa presentation in men of African ancestry.

**Supplementary Information:**

The online version contains supplementary material available at 10.1186/s13073-022-01096-w.

## Background

Prostate cancer (PCa) is a significant health burden for men of African ancestry. In the USA, African American men are more likely to present with aggressive disease [1], with mortality rates 2.3- (≥ 65 years) and 3.1-fold (< 65 years) greater than men of European ancestry and as much as 5-fold greater than men of Asian ancestry [2]. Within sub-Saharan Africa, mortality rates are double global averages, reaching as much as 2.7-fold for southern Africa [3]. Previously, we have shown that southern Africans have a 2.1-fold greater risk for aggressive PCa at presentation than reported for African Americans (adjusting for age) [4]. Hypothesising that both genetic and non-genetic factors are driving ethnic disparity, we speculate that these differences are likely to be evident in the landscape of variants acquired during tumour growth. Still, little data is available for Africa. In an attempt to close this gap, we previously reported a 1.13 to 1.8-fold increase in tumour mutational burden (TMB), defined by the total number of somatic single nucleotide variants (SNVs) and small insertions and deletions (indels; length <50 bases) per megabase (Mb) of whole genome, in predominantly treatment-naïve high-risk (Gleason score ≥8) prostate tumours derived from men of southern African *versus* European ancestry [5, 6]. Observing a larger range of TMB in tumours derived from African (0.031 to 170.445 mutations/Mb) compared to European patients (0.015 to 2.145 mutations/Mb), we found mutational types to be strongly correlated and, as such, tumours harbouring the greatest number of structural variations (SVs; length ≥ 50 bases) were more likely to be derived from men of African ancestry [6]. To the best of our knowledge, no study has performed an in-depth interrogation of the range and type of SV that may be contributing to aggressive PCa presentation in patients from any region within sub-Saharan Africa.

Investigating SVs is critical for comprehensively describing and analysing the genomic burden of PCa [7, 8]. Notably, the most common somatic alteration in PCa involves an intrachromosomal translocation or 3Mb deletion on chromosome 21, resulting in fusion of the androgen-responsive gene *TMPRSS2* and members of the E26 transformation-specific (ETS) transcription factor family [9]. *TMPRSS2-ERG* gene fusions are common to roughly 50% of prostate tumours from men of European ancestry [10], dropping to 25% in African Americans [11] and 13% in Black men from South Africa [12]. We speculate that tumours from African patients may present with a distinct SV landscape and associated fusion oncogenes. In addition to simple deletions, insertions, duplications, inversions and inter- or intra-chromosomal translocations, SVs appear to be uniquely complex in PCa, demonstrated by the phenomenon of chromoplexy, involving an abundance of interdependently occurring translocations and deletions [7]. While we reported chromoplexy to be more frequent in tumours from European (38%) *versus* African (33%) patients, conversely, African-derived tumours were more likely to present with a larger number of inter-chromosomal chained fusions (1-6 *versus* 1-2) [6].

Expanding on our earlier work to generate through deep whole genome sequencing (WGS) a tumour mutational profile for PCa in sub-Saharan Africa, describing a new molecular taxonomy [6], in this study we provide an in-depth interrogation for the type, frequency, distribution, ethnic disparities and associated clinical impact of the largely overlooked somatic SVs. Specifically, we interrogated the landscape of somatic SVs in treatment-naïve primary prostate tumours derived from 180 African *versus* European ancestral patients, with a bias towards high-risk disease. Including 114 African ancestral men from southern Africa (South Africa) makes this study, to the best of our knowledge, the largest of its kind for the region and greater sub-Saharan Africa. The inclusion of Europeans (predominantly Australians) allowed for direct comparison for prevalence of SVs in types and genomic regions using a single experimental and analytical pipeline. Ultimately, we elucidated the potential role of somatic SVs contributing to aggressive PCa in men of African ancestry, which may at least in part explain the significant ethnic-based disparity.

## Methods

### Patient clinicopathology and ancestry assignment

In this study, 180 clinicopathologically confirmed PCa patients were recruited from South Africa (*n*=120), Australia (*n*=53) and Brazil (*n*=7). As previously described [6], South African men were recruited at the time of diagnosis from Southern African Prostate Cancer Study (SAPCS) participating urology clinics located within the greater Limpopo and Gauteng provinces [4]. All patients were treatment naïve at time of sampling. Australians attending the Prostate Cancer Clinic at St Vincent’s Hospital in Sydney, and Brazilians attending a participating academic clinic in the greater State of Rio Grande do Sul, were recruited at the time of radical prostatectomy. A single Australian patient (15178) had received one-month-long Ozurdex therapy prior to surgery, while only two Brazilian patients could be confirmed as treatment naïve prior to sampling. Three patients recruited from South Africa with no clinicopathological evidence for PCa and described in a previous study [6] were excluded. Overall, our study was biased towards advanced disease presentation, defined as a Gleason score ≥ 8 (138/180, 76.7%).

Germline WGS data provides clarification of genetic ancestral contributions [6], with ancestry informativeness assigned based on 7,472,833 biallelic SNVs across the genome using the population analysis tool fastSTRUCTURE v1.0 [13]. Consequently, 115 patients (63.9%) are African ancestral (114 South African, 1 Brazilian), with >78% African genetic contribution and 111 showing no non-African contributions; 61 patients (33.9%) are European ancestral (53 Australian, 4 South African, 4 Brazilian), of which five showed minimal Asian genetic contributions (3.3–26.3%) and a single patient minimal African ancestral contribution (15.7%) and four patients were classified ancestrally as admixed (2 South African, 2 Brazilian), demonstrating large African (31-63%) and European (37–59%) genetic fractions (Additional file 1: Table S1).

Clinicopathological features of the study participants defined by ancestry show a 5-year greater mean age and 6-fold greater prostate-specific antigen (PSA) level at presentation for African *versus* European patients (Additional file 1: Table S1). Our cohort concurs with our previous findings for significantly elevated PSA levels in Black men from South Africa, irrespective of PCa status, as well as an overall older age at presentation [4]. However, on pathological analysis, for the 138 (77%) cases presenting with high-risk Gleason score ≥8 PCa, 70.4% (81/115) are of African and 86.8% (53/61) of European ancestry.

### WGS data generation

As previously described [6], all samples underwent a single technical pipeline from DNA extraction to data generation. DNA was extracted from fresh-frozen prostate tumour tissues, derived either from biopsy core at diagnosis or from surgical tissue, as well as matched blood samples, using DNeasy blood and tissue kit protocol (Qiagen, Maryland). WGS was performed with 2×150 cycle paired-end mode on Illumina HiSeq X Ten (21 cases) or NovoSeq (159 cases) instruments at the Kinghorn Centre for Clinical Genomics (Garvan Institute of Medical Research, Australia). Following the BROAD’s best practice recommendations for “data pre-processing for variant discovery”, scalable FASTQ-to-BAM (v2.0) workflow with default settings was used to align sample sequencing reads to the GRCh38 reference genome with alternative contigs [14]. The mean depth of coverage for the tumour and matched normal samples were 90× (range 28–139×) and 46× (range 30–97×), respectively. Tumour purities ranged from 13 to 88% (mean of 48%), as estimated by Sequenza (v2.1.2) [15].

### Somatic structural variant calling and gene annotation

Somatic SVs were called using Manta (v1.6.0) [16] and GRIDSS (v2.8.3) [17, 18] for each pair of tumour and normal samples. High-confidence SV calls from Manta were defined as those reported with ‘PASS’ in the VCF FILTER field in the output VCF file. SV types reported by Manta include deletions, tandem duplications, inversions, insertions and adjacent breakend (BND) for a fusion junction in an inter-chromosomal rearranged genome. Pairs of BND were annotated as inter-chromosomal translocations. High-confidence SV calls from GRIDSS were obtained using GRIDSS accompanied R script (gridss_somatic_filter.R). GRIDSS reports BND for all fusion junctions resulting from any SV event. Simple SV types, defined as deletions, duplications, insertions and inversions, were assigned using the accompanied R script: simple-event-annotation.R, while inter-chromosomal BND pairs were further annotated as translocation, in the same way as Manta. When integrating call sets from Manta and GRIDSS, two SV calls were considered as concordant if they were reported as high-confidence by one of the two callers (Additional file 1: Fig. S1) and have matching SV type and reported breakpoint positions within 5bp of each other. This new filtering method is able to overcome the limitation of the high-confidence definition of different SV callers and rescue more false negatives [19] and provides a more comprehensive SV call set compared to our earlier study [6]. Germline SVs were called by Manta (v1.6.0) with filtration of ‘PASS’ in FILTER field in the VCF file.

Gene annotation of all SV breakpoints was performed using the Ensembl human gene annotation (GRCh38 assembly, release 99). SV BND was annotated as ‘interrupting’ if it was located within a gene region. A SV event is classified as a gene fusion if both BNDs interrupt two different genes. Annotation of exons of SV breakpoints in *TMPRSS2-ERG* fusion-positive samples was based on the exon regions (exonStarts and exonEnds) for all transcripts in the UCSC Table *refFlat* [last updated 08/17/2020] from the NCBI RefSeq track for GRCh38. There are two transcripts of *TMPRSS2*, each with 14 exon regions and 10 transcripts of *ERG*, each with a different number of exon regions [5–12]. For each transcript of *ERG*, the upstream exon to the SV breakpoint interrupting *ERG* was identified. For each transcript of *TMPRSS2*, the downstream exon to the SV breakpoint interrupting *TMPRSS2* was identified. This process was done for all combinations of transcripts of *TMPRSS2* and *ERG* genes and all SV breakpoints in *TMPRSS2-ERG* fusion-positive samples.

### Germline and somatic mutation (SNVs and indels) calling and annotation

Following the BROAD’s best practice recommendations for “germline short variant discovery (SNPs + Indels)” and “somatic short variant discovery (SNVs + Indels)”, small germline and somatic mutations (SNVs and indels) were called using the scalable Germline-ShortV v.1.0 [20] and Somatic-ShortV v.1.0 workflows [21], respectively. Both germline and somatic variants were annotated using annovar (version 2019Oct24) with the RefSeq gene database (build version Hg38) [22].

### Copy number variation calling

Somatic copy number variation (CNV) with discrete copy number segments were determined using the copy number calling pipeline of CNVKit [23]. These were further examined using GISTIC v2.0.23 [24] to identify CNV at the gene level. CN gains (amplifications) or losses (deletions) per gene were determined based on CN values estimated as 2 or −2 respectively from GISTIC output CN values (all_threshold.by_genes.txt). CN values were estimated as ±2 if exceeding the high-level thresholds and ±1 if exceeding the low-level thresholds, but not the high-level thresholds [24]. For CN gains and losses, the low-level threshold values are 0.1 and −0.1 respectively, while the high-level thresholds were calculated on a sample-by-sample basis by GISTIC.

### Recurrent mutation in hyper-SV mutated tumours

Recurrent somatic mutations (SNVs, indels and CNVs) in hyper-SV mutated tumours were examined for 631 previously described PCa driver genes [6]. Logistic regression was used to test the null hypothesis of no correlation between the total count (and relative frequency) of each SV type and most recurrent mutated genes, based on variant types of SNVs, indels and CNVs. *P*-values were adjusted for multiple testing correction using the Benjamini-Hochberg method.

### Gene biallelic inactivation classification

We examined three genetic inactivation types defined in the study of Campbell et al. [25], including ‘Loss’ (somatic or germline deletions), ‘Break’ (somatic or germline SVs) and ‘Mutation’ (somatic or germline SNVs) for *BRCA2* in hyper-deletion and *CDK12* in hyper-duplicated tumours. For a gene G with A and B alleles (G^A/B^), four classes of biallelic inactivation of both alleles (G^−/−^) were defined as (1) Loss/Mutation, loss of the A allele and nonsynonymous driver mutation of the B allele; (2) Loss/Loss, two deletions overlapping an exon and CN derived allele count is 0 both for A and B alleles; (3) Loss/Break, loss of the A allele and SVs where one or both breakpoints interrupting an exon of B allele; and (4) Mutation/Mutation, a nonsynonymous germline SNV and a nonsynonymous somatic SNV of the same gene [25].

## Results

### Spectrum of somatic structural variant types

**Fig. 1:**
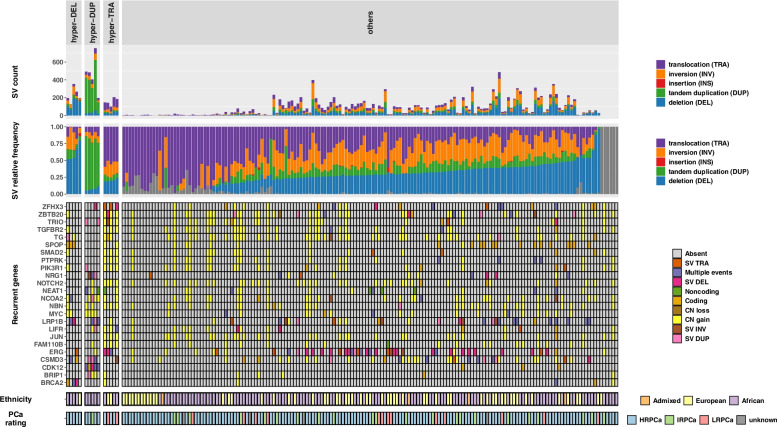
The spectrum of somatic structural variations (SVs) based on type classification. Top two panels are the total count and relative frequency of each SV type. Samples in the horizontal axis are placed in the order of increasing relative frequency of deletions. The “Recurrent genes” heatmap presents the 24 most recurrently mutated genes, each with at least three samples in each hyper-SV group. Coding, SNVs/indels in coding region; Noncoding, SNVs/indels in noncoding region; HRPCa, high-risk PCa; IRPCa, intermediate-risk PCa; LRPCa, low-risk PCa

Defined by ethnicity and PCa risk at diagnosis or surgery, we observed large variability in the number of somatic SVs (range 0–754) per tumour (Fig. 1). In their simplest form, deletions and inversions were found to be the most common SV types, while consistent with other PCa studies, we observed a low frequency of insertion events (Additional file 1: Fig. S2). Only 15 tumours (8.3%) presented with at least one insertion, which may be due to the limitations of insertion detection using short-read next generation sequencing data [19]. We found duplication events to have the greatest variability by count and relative frequency among the ethnic groups, representing a 2.5- and 1.6-fold increase in tumours from African *versus* European patients, respectively. While not observed in our study, Quigley et al. reported a significant association between biallelic inactivating alterations in *TP53* and the frequency of inversions in mCRPC [26]. Here we found the relative frequency of inversions to be significantly associated with *SPOP* mutations (adjusted *p*-value = 0.04).

Furthermore, we define hyper-SV mutated tumours as having at least 100 (average SVs per tumour in this cohort) total count of SVs with at least 50% dominated by a single SV type. As such, we identified five hyper-duplicated (KAL070, N0067, SMU087, UP2050 and UP2133), five hyper-deleted (BRA08, KAL0013, UP2003, UP2103, UP2396) and five hyper-translocated (UP2187, 5656, 12596, UP2267 and SMU142) tumours, their CIRCOS plots are shown in Fig. S3 (Additional file 1). Hyper-SV tumours were notably biased towards tumours derived from men of African ancestry (Fig. 1), with the hyper-duplicated genomes African-specific (5/5), the hyper-deleted African-biased (4/5) and the hyper-translocated observed in tumours from both African (3) and European (2) patients. To the best of our knowledge, no study has reported the hyper-translocated PCa subtype to date [26–29].

In non-African studies, the hyper-duplicated mutation subtype has been reported in 3% and 7% of metastatic castration-resistant PCa (mCRPC) by Quigley et al. (*n* = 101) [26] and Van Dessel et al. (*n* = 197) respectively [29] and in 22% and 20% of smaller cohort studies by Viswanathan et al. (*n* = 23) [30] and Wedge et al. (*n* = 10) respectively [27]. Conversely, this subtype was notably absent in localised PCa from studies including The Cancer Genome Atlas (TCGA) WGS data (*n* = 20) [31], Fraser et al. (*n* = 200) [28] and Wedge et al. (*n* = 92) [27]. Importantly, while sourced from primary tissue, the status of metastatic seeding is unknown for our African patients. Furthermore, enrichment of duplications has previously been associated with bi-allelic *CDK12* inactivation (*CDK12*^−/−^) in mCRPC [26, 29, 30]. Here we found the relative frequency of duplications per tumour to be significantly correlated with *CDK12* mutation (adjusted *p*-value = 0.001) with four hyper-duplicated tumours found to be *CDK12*^−/−^ (Fig. 1 and Additional file 1: Table S2). Although tumour SMU087 has both somatic CN loss and deletion detected on *CDK12*, it did not satisfy the criteria for assessment of *CDK12* biallelic loss. In addition, we found *MYC* CN gains to be significantly associated with increased relative frequency of duplication per tumour (adjusted *p*-value = 0.03), with four of the hyper-duplicated tumours presenting with *MYC* CN gains (Fig. 1).

Enrichment for deletions (<100kbp) in non-African studies has been found to be associated with bi-allelic *BRCA2* mutation (*BRCA2*^−/−^) in mCRPC [26, 29]. In our study, we observed *BRCA2*^-/-^ in three hyper-deleted tumours from African patients. While the two remaining hyper-deleted tumours, BRA08 (European) and KAL0013 (African), showed no *BRCA2* loss, two or more nonsynonymous germline *BRCA2* mutations were observed for each patient (Additional file 1: Table S3), although defined by ClinVar [December 2020] [32] as ‘benign’ or of ‘uncertain significance’. This suggests the hyper-deleted signature observed in these two patients is either unrelated to biallelic *BRCA2* loss or the clinical significance of these two germline SNVs is under-recognised. *BRCA2* mutation was not found statistically associated with count of deletions in this study. However, we found the count of deletions per tumour to be significantly associated with the presence of somatic *SPOP* mutations (adjusted *p*-value = 0.005, Fig. 1), which presented in three hyper-deleted tumours from African patients, including the single none-*BRCA2*^*-/-*^ African-derived tumour (patient KAL0013)*.*

### Spectrum of somatic structural variant breakpoints

**Fig. 2:**
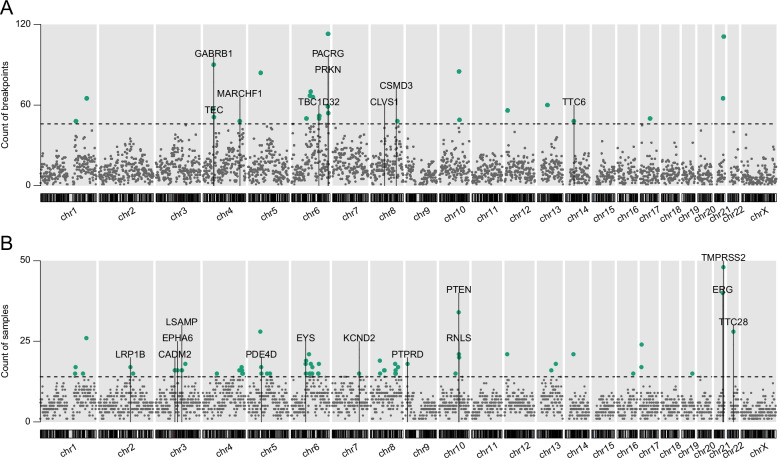
Genome-wide structural variation (SV) frequency across all 180 samples. Dots show the number of SV breakpoints (**A**) and the number of samples with SVs (**B**) within 1 Mbp windows, with hotspot regions (>3 SD from mean, threshold shown as dashed horizontal line) highlighted as green. Hotspots where genes are interrupted by >50% breakpoints or recurrent in >50% genomes are labelled

We identify SV hotspots based on (i) the total number of SV breakpoints and (ii) the number of samples with at least one SV breakpoint for each 1 Mb non-overlapping bin across the genome. Overall, each bin contained 10 ± 7.4 (median ± MAD) breakpoints from 6.0 ± 3.0 samples. SV hotspots were then defined as genomic regions most frequently (> Q_3_ + *k* × (Q_3_ − Q_1_)) affected by SV breakpoints, either in the same genome or recurrent across genomes (Additional file 1: Fig. S4). Based on Tukey’s fences approach, *k* = 1.5 was used to find outliers in sample count (Additional file 1: Fig. S4B), while more stringent *k* = 3 was used to define outliers for SV breakpoints count, considering clustered SV breakpoints such as chromothripsis can be attained in a single tumour (Additional file 1: Fig. S4A). In summary, 74 genomic bins (from a total of 2833) were identified as SV hotspots (Fig. 2), and 13 hotspots were found to be both frequently affected by SV breakpoints (>46 breakpoints) and recurrent among samples (>14 samples). Of all 74 hotspots, 26 presented with >50% SV breakpoint interrupting a single gene or with the single gene interruption recurrent in >50% genomes. *ERG*, *PTEN*, *CSMD3* and *LRP1B* were previously identified as driver genes associated with PCa using this sample data source and PCAWG cohorts [6], while our new method highlighted 18 additional potential driver genes, including *GABRB1*, *CLVS1*, *RNLS*, *TMPRSS2*, *TTC28*, *EYS*, *TTC6*, *PTPRD*, *PRKN*, *PACRG*, *TBC1D32*, *CADM2*, *LSAMP*, *MARCHF1*, *PDE4D*, *KCND2*, *EPHA6* and *TEC*. Among the gene candidates, *EYS*, *PTPRD*, *PRKN*, *CSMD3*, *CADM2*, *LSAMP* and *PDE4D* are larger than the defined genomic bin (1 Mbp) and as such we cannot exclude for their co-location with the SVs being a chance event. Additionally, we recognise that *PACRG*, *LRP1B* and *PDE4D* are putative fragile sites [33]. Additionally, we observe three hotspots (chr6: 66–67 Mbp, chr6: 94–95 Mbp, chr5: 85–86 Mbp and chr13: 64–65 Mbp) with <5% breakpoints overlapping gene regions, which may indicate a different mechanism in promoting PCa.

**Fig. 3:**
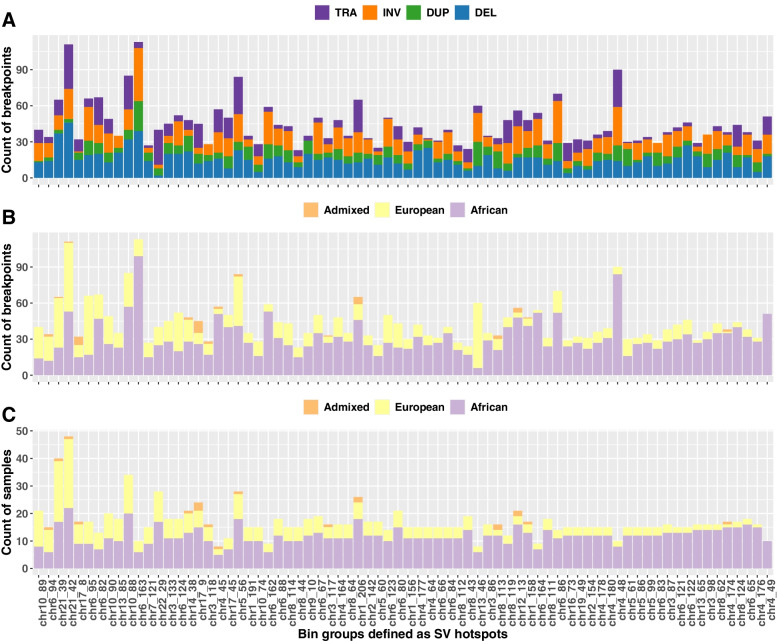
Structural variation (SV) types and ethnic groups in SV hotspot regions. The count of SV breakpoints per SV hotspot, coloured by SV types (**A**) and ethnic groups (**B**). The count of samples per SV hotspot coloured by ethnic groups (**C**). SV hotspots represented by 1 Mbp non-overlapping bin groups are ordered by the percentage of samples of African ancestry

All identified SV hotspots included multiple SV types (Fig. 3A). 12 hotspots contained >50% deletions of all SV events within the bin, including the *ERG* gene region (chr21: 38–39 Mbp). One hotspot (chr6: 66–67 Mbp) includes more inversion events (52%) and two hotspots (chr16: 72–73 Mbp and chr22: 28–29 Mbp) include more translocation events (52% and 73%, respectively). The hotspot chr8: 43–44 Mbp was found with an even distribution of deletions, duplications, inversions, and translocations of around 20% for each SV type.

Taking ethnicity into consideration, eight hotspots were found to have elevated number of SV breakpoints (≥90%) in patients representing a single ancestry, of which seven were specific to Africans (Fig. 3B). Notably, the single European-specific hotspot (chr13: 45-46 Mbp) was driven by a large number of SVs in a single tumour from a European patient. However, when considering patient count, we observed 6/8 tumours to be African-derived (Fig. 3C). Overall, 65/74 SV hotspots have > 50% breakpoints observed in >50% tumours of African ancestry. In hotspots chr4: 48–49 Mbp and chr4:175–176 Mbp, more than 90% of tumours are derived from African patients and significantly associated with African ancestry (*p*-value = 0.04 and 0.03 respectively by Chi-squared test).

Although *TEC* was found to be a candidate driver gene interrupted by 55% breakpoints in the African-dominant hotspot chr4: 48–49 Mbp, only 2/10 tumours have *TEC* disruption. Among the 22 candidate driver genes found in SV hotspots, previously reported PCa-related gene *ERG* interruption at hotspot chr21: 38–39 Mbp (37/40 tumours) was biased towards European-derived tumours (21/37), representing 34.4% and 13.0% of tumours from European and African patients, respectively. Conversely, we found the previously identified PCa driver gene *LRP1B* (12/17) and new candidate genes *TTC28* (16/27), *CADM2* (12/16), *LSAMP* (11/16), *EYS* (16/18), *PTPRD* (12/18), *PACRG* (5/6) and *PDE4D* (12/17) to be predominately interrupted in tumours from African patients.

### Gene fusions: SV types and breakpoint clustering

Through investigation of gene regions impacted by SV BND pairs, we identified 6,617 gene fusions, in which 134 are recurrent in two genomes, 13 in three genomes and 6 in four or more genomes (Additional file 2: Table S4 and Table S5). 33 gene fusions were previously reported for PCa, including two of the top six recurrent gene fusions *ZBTB20-LSAMP* (4 tumours of which three are African-derived), and the well-established PCa fusion gene *TMPRSS2-ERG* (31 tumours) [34]. Among the novel gene fusions identified, 144 are recurrent (two or more) in tumours from African patients (Additional file 2: Table S4). The top novel African-associated recurrent gene fusions include *AC016822.1-PCDH15* (4/4), *AC098650.1-RBMS3* (3/3), *AC117473.1-EPHA6* (3/3), *AL513166.1-FPGT-TNNI3K* (3/3), *AL513166.1-TNNI3K* (3/3), *CASC19-PCAT1* (5/5), *DPYD-DPYD-AS1* (3/3), *PRKN-PACRG* (3/4) and *SATB1-TBC1D5* (3/3). Additionally, we observed 35 intra-chromosomal SVs within the African-derived *PRKN-PACRG* positive tumour N0081.

**Fig. 4:**
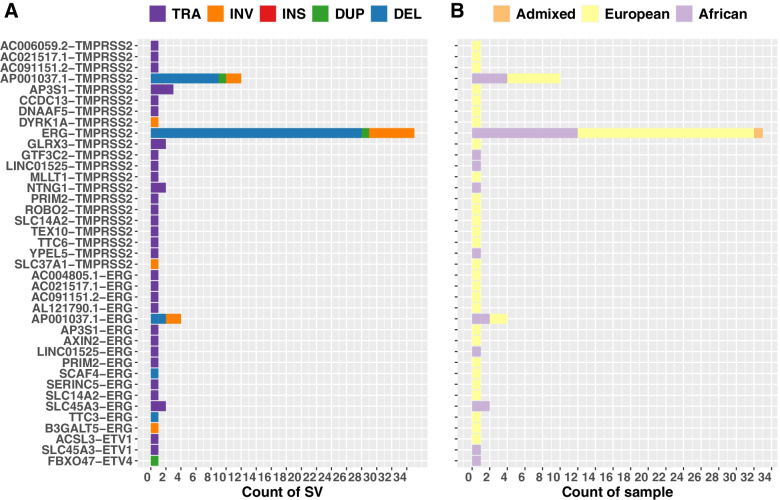
*TMPRSS2* and *ETS* family gene fusions. **A** shows the count of SVs involved with colour representing SV types and **B** shows the count of the sample having the corresponding gene fusion, with colour representing the ethnic groups. Abbreviations: SV, structural variation; TRA, translocation; INV, inversion; INS, insertion; DUP, duplication; DEL, deletion

Taking a closer look at *TMPRSS2-ERG* and their alternative partners, as well as all previously reported PCa relevant ETS family members, namely *ETV1*, *ETV4* and *ETV5* [10], we identified besides *TMPRSS2-ERG*, 20 *TMPRSS2*, 15 *ERG*, 2 *ETV1* and 1 *ETV4* partners, largely driven by translocations [35] and to a lesser extent by deletion [13], inversion [7] and duplication [2] events (Fig. 4A). While 23 were European-specific, 10 were African-derived (Fig. 4B). *SLC45A3-ERG*, identified in 2/115 (1.7%) tumours from African patients, after *TMPRSS2-ERG* is the second most common fusion event in *ERG* positive PCa tumours [35], while *SLC45A3-ETV1* fusion has also been reported [36]. In our tumours from African patients, we identified *FBXO47* as a novel PCa partner to *ETV4* and *LINC01525* to *ERG*. African-specific novel PCa partners to *TMPRSS2* include *GTF3C2*, *LINC01525*, *NTNG1* and *YPEL5*.

Further investigation of *TMPRSS2-ERG* fusion identified two additional tumours harbouring large (around 2.8 Mbp) deletion events, with BNDs 73,116 bp and 33,796 bp downstream of *ERG*, respectively. Of the 33 *TMPRSS2-ERG* gene fusion-positive patients, 20 are European (33% of 61), 12 African (10% of 115) and one of admixed African-European ancestry. The lower percentages observed across our study coincides with previously reported ethnic disparities [11, 12], as well as the increased presence of this fusion event reported for lower-grade tumours [37]. Previously attributed to an interstitial deletion or an insertional chromosomal rearrangements [38], of the 33 tumours identified as *TMPRSS2-ERG* fusion-positive in this study, 16 are the result of a single deletion event, two present with a deletion and each additionally with two matching translocations (2 pairs of BNDs), indicating retention of the interstitial region (Additional file 1: Fig. S5), and 10 present with a deletion with additional overlapping SVs. Of the remaining tumours, two were the result of inversion events (European patients 15126 and 5902), one from a duplication (African patient TSH005), while a single tumour from a European (BRA10) and African (UP2103) patient involved four and seven overlapping SVs, respectively. For BRA10, the multi-event *TMPRSS2-ERG* fusions included: one deletion between *TTC3* and *ERG*, one inversion on *ERG*, one inversion between *ERG* and *TMPRSS2* and one inversion between *TMPRSS2* and *SLC37A1*. For UP2103: two translocations between *NTNG1* and *TMPRSS2*, one translocation between *LINC01525* and *ERG*, one translocation between *LINC01525* and *TMPRSS2*, one inversion between *ERG* and *TMPRSS2* and two inversions on *TMPRSS2*. It is unclear if the fusion is the result of the inversion or multiple SVs found in BRA10 and UP2103.

**Fig. 5:**
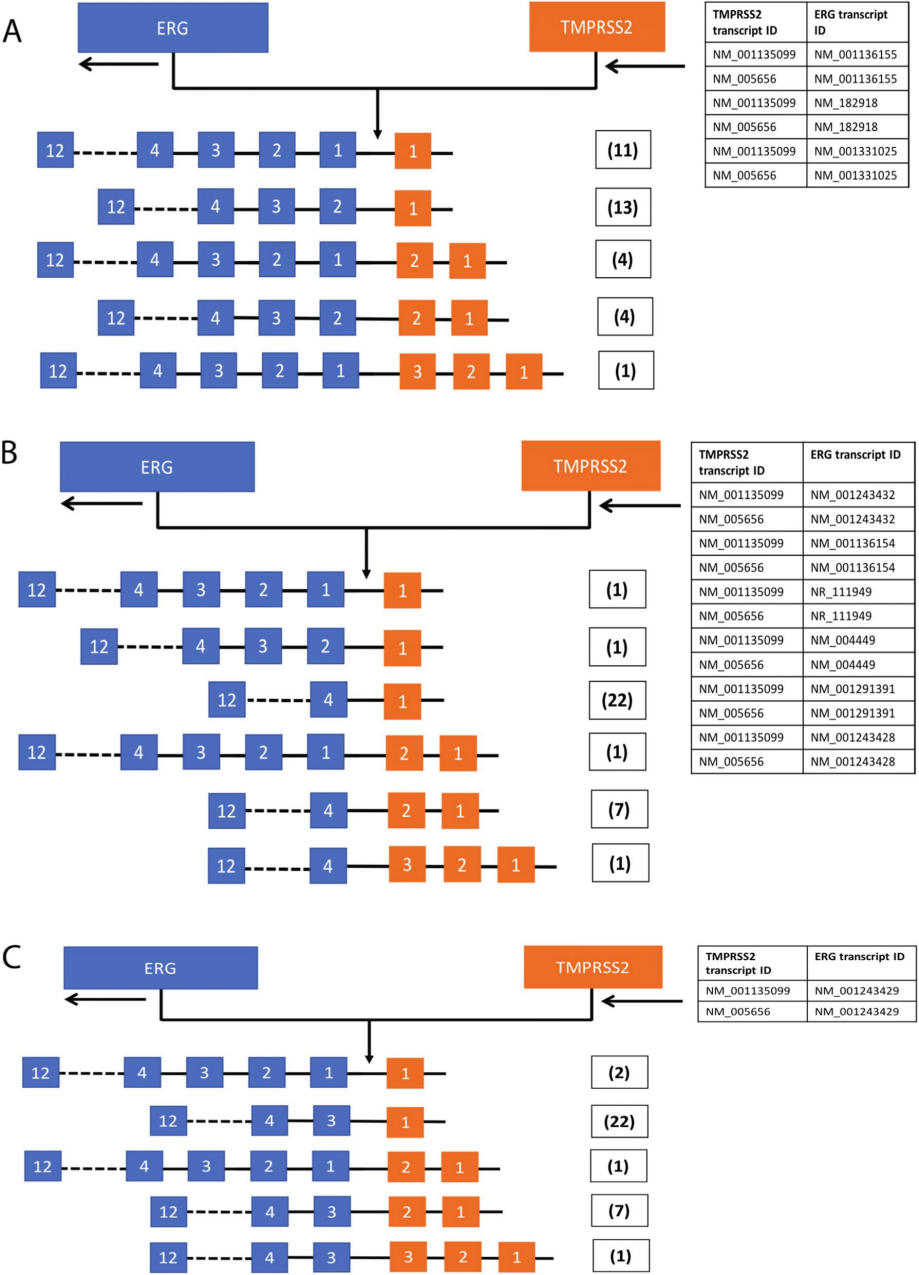
Spectrum of gene fusion junctions. **A**–**C** panels show the three forms of structural variation (SV) breakpoint clusters based on different transcripts of *TMPRSS2* and *ERG*, shown in the top right. The number of samples with breakpoint in different exon positions of *TMPRSS2-ERG* fusion junction is shown in brackets

Investigating if SV breakpoints involved in *TMPRSS2-ERG* fusions cluster in any specific genomic position, we found breakpoints on *TMPRSS2* (2 transcripts) to be clustered predominantly 3′ of exon 1 or 2, while breakpoints on *ERG* (10 transcripts) clustered 5′ of exon 3 or 4 (Fig. 5). These observations are consistent with previous findings using RNA expression data [12, 38], while UP2103, UP2089 and UP2093 were previously included in a targeted RNA sequencing study aimed at defining the exact *TMPRSS2-ERG* fusion transcript junction coordinates [12]. The latter study detected three or four *TMPRSS2-ERG* fusion transcript junctions (isoforms) with different coordinates from all of the three samples, while we identified a single deletion in UP2089 and UP2093 and a single inversion in UP2103. Thus, these studies concur that a single genomic fusion can result in multiple fusion transcripts or isoforms.

## Discussion

To our knowledge, this is the first study to investigate the potential role of SVs in significant risk for aggressive PCa observed for men of African ancestry from sub-Saharan Africa, specifically southern Africa. Through direct ethnic-based comparative analysis using a single technical and informatic pipeline, we report a higher variability of somatic SVs for tumours from African (0–754) *versus* European (0–398) patients. Comparing the prevalence of SVs in types between tumours from African and European patients, duplication had the greatest difference among the ethnic groups, showing a 2.5- and 1.6-fold increase in African *versus* European-derived tumours in its average count and relative frequency, respectively. Hyper-SV mutated tumours were largely restricted to African patients (12/15). While a study of 20 metastatic tumour types, including PCa, found stomach and oesophageal tumours to be highly enriched for translocations [39], this is the first report of a hyper-translocated PCa subtype. Additionally, this is the first study to identify hyper-duplicated and hyper-deleted non-treated primary tumours rather than mCRPC. While the metastatic status of the African patients is unknown, our study suggests that hyper-SV is likely an indicator of aggressive disease rather than a consequence of treatment response. Confirming a link between *CDK12*^-/-^ and hyper-duplicated (4/5 tumours), we identify an additional association with *MYC* CN gain. While our study concurs with *BRCA2*^-/-^ in hyper-deleted tumours (3/5), here we report further association with *SPOP* mutation.

SVs have previously been shown to be non-randomly distributed across cancer genomes [40], implying that SVs at certain loci may drive the expansion of some cancer clones. Applying an independent SV hotspot analysis approach, 74 hotspots were identified based on the number of breakpoints or recurrent tumours in each genomic window, revealing 18 new potential driver genes. Investigating the prevalence of SV hotspots in ethnic groups, hotspots chr4: 48-49 Mbp and chr4:175-176 Mbp were predominantly found in African-derived tumours. In addition, we found *ERG* as European-derived tumour-related driver and five new African-derived tumour-related driver genes, including *TTC28*, *CADM2*, *LSAMP, EYS*, *PACRG*, *PDE4D* and *PTPRD.* Large number of inter-chromosomal translocation inactivating *TTC28* has been reported in colorectal cancer [41]. *CADM2* acts as a tumour suppressor in renal cell carcinoma [42], prostate cancer [43] and hepatocellular carcinoma [44], and has been reported promoting brain metastasis in lung cancer and proposed as a potential molecular target [45]. Recurrent deletions of the *LSAMP* locus have been reported in tumours from African American men, identifying an African-specific aggressive PCa subset [46]. *PACRG* has previously been reported to be associated with poor prognosis of renal cell carcinoma [47]. The high expression of *PDE4D* has been reported to be associated with aggressive disease in multiple cancers, with therapeutic potential reported for pancreatic ductal adenocarcinoma [48], tamoxifen-resistant ER-positive breast cancer [49], lung cancer [50] and colon cancer [51]. In PCa, *PDE4D* has been implicated as proliferation-promoting factor and proposed as a biomarker and potential drug target [52, 53]. *PTPRD* was classified as a tumour suppressor gene, which has been reported to be highly mutated and correlated to the disease progression in colon [54] and gastric cancers [55] and found deleted in multiple types of cancers [56]. However, *PTPRD* has been reported as a significantly low-frequency mutated gene in PCa [57], indicating SV may be an alternate variant type activating *PTPRD* in African patients.

Investigation of gene fusions caused by SVs identified; the well-established PCa fusion gene *TMPRSS2-ERG* in 10% and 33% of tumours from African and European patients, respectively; the previously reported African-specific *ZBTB20-LSAMP*; and nine novel African-associated fusions. *LSAMP* was identified as a new potential driver gene in this study; other studies also reported a significantly higher number of inter-chromosomal rearrangements and exclusive association of *LSAMP* deletion/rearrangement for African American tumours, including *ZBTB20-LSAMP* gene fusion [46]. Investigating *TMPRSS2-ERG* and their alternative partners revealed six (out of 29) African-derived fusions with novel PCa partners to *ETV4, ERG* and *TMPRSS2*, including *FBXO7*, *LINC01525, GTF3C2* and *NTNG1.* Classified as a potential tumour suppressor gene, disruption of *FBXO47* has been reported in breast, ovarian and renal cancers [58, 59], while to the best of our knowledge this is the first report of *LINCO1525* disruption in any cancer. *GTF3C2* is one member of the general transcription factor III family, which has been reported as a prognostic factor in liver cancer [60]. *NTNG1* belongs to the family of netrins, with an elevated level of *NTNG1* reported to result in cisplatin resistance in ovarian cancer [61]. *NTNG1* mutation has also been associated with poor prognosis in colorectal [62] and pancreatic cancer [63]. A study of more than 10,000 samples across more than 30 tumour types found *NTNG1* to have the highest mutation rate in the netrin family observing *NTNG1* fusion transcripts in multiple cancers, including breast, lung and skin [64]. Increased expression of *YPEL5*, coding for a member of the carboxy-terminal to LisH (CTLH) complex, has been reported in erlotinib-treated EGFR-mutant non-small cell lung cancer [65], while recurrent *YPEL5-PPP1CB* fusion has been reported for chronic lymphocytic leukaemia [66].

Deletion and translocation have been reported to cause 5’ untranslated region of *TMPRSS2* to be fused with *ERG* on the same directions of the two genes, resulting in chimeric proteins [38]. With further investigation of *TMPRSS2-ERG* gene fusion-positive tumours, 17/33 of them harbour multiple SV events of different SV types in addition to deletion and translocation events. Furthermore, we found inversions with BND interrupting *TMPRSS2* and *ERG* genes in three tumours from European patients and one RNA-seq validated tumour from the African patient UP2103 [12]. The inversion resulted in *TMPRSS2* and *ERG* genes fused in opposing coding strand directions, which may result in the formation of a chimeric transcript with a similar role to an antisense RNA [67]. Overall, our study demonstrates the complexity of SV events resulting in *TMPRSS2-ERG* fusion that cannot be attributed to simple DNA loss or translocation.

The use of short-read sequencing is a potential limitation of this study. We have used high-coverage WGS and employed the best-practise SV calling and filtering approach to achieve the balance of detection sensitivity and precision, but may have overlooked a fraction of real SVs, in particular the known to be ‘difficult-to-detect’ insertions and/or those present at the sub-clonal level [19]. Future studies using long-read sequencing may reveal a greater SV burden and additional hotspots. In addition, the discovered novel oncogenic drivers in this study have yet to be validated. The better prognosis and treatment for African PCa patients can benefit from further African-relevant validation and functional studies of the discovered hotspots and candidate drivers in this study.

## Conclusions

As a hallmark feature of its genome, SV is a major contributor to the development and progression of PCa by gene disruption and enabling genomic instability. Here we have described in a first-of-its-kind study the spectrum of simple SV types and SV-derived hotspots, including novel oncogenic drivers and gene fusions specific to African patients that may explain, at least in part, the observed disparity in PCa aggressiveness observed for men of African *versus* European ancestry. The identification of novel African-specific prognostic, including *PTPRD, LSAMP, PACRG*, *FBXO7*, *GTF3C2* and *NTNG1*, and therapeutic targets, including *CADM2*, *PDE4D* and *YPEL5*, emphasises the need for both African inclusion and SV interrogation to reduce advanced PCa ethnic disparity through tailored clinical management.

## Supplementary Information

**Additional file 1: Figure S1.** Concordant SV call generation from Manta and GRIDSS. **Figure S2.** Summary of SVs in each type, compared to other studies. **Figure S3.** CIRCOS plot of hyper-SV mutated tumours. **Figure S4.** The spread of SV breakpoints and samples in each 1 Mbp genomic bin. **Figure S5.** *TMPRSS2-ERG* fusion with interstitial region retention. **Table S1.** Clinical and pathological characteristics of 180 prostate cancer patients included in this study. **Table S2.** Biallelic assessment of *CDK12* in hyper-duplicated samples. **Table S3.** Biallelic assessment of *BRCA2* in hyper-deleted samples.

**Additional file 2: Table S4.** Summary of gene fusions identified from SVs. **Table S5.** SV calls resulting in gene fusions.

## Data Availability

The datasets analysed in this study were obtained and accessible through Jaratlerdsiri et al [6], with sequence data deposited in the European Genome-Phenome Archive (EGA; https://ega-archive.org) under overarching accession EGAS00001006425 and including the Southern African Prostate Cancer Study (SAPCS) Dataset (EGAD00001009067) and Garvan/St Vincent’s Prostate Cancer Database (EGAD00001009066). The computational code used to analyse SV subtypes, SV hotspots and gene fusions is available on GitHub [68].

## Abbreviations

PCa: Prostate cancer
SV: Structural variation
TMB: Tumour mutational burden
SNV: Single nucleotide variants
indels: Small insertions and deletions
Mb: Megabase
ETS: E26 transformation-specific
DEL: Deletion
INS: Insertion
DUP: Duplication
INV: Inversion
TRA: Translocation
WGS: Whole genome sequencing
PSA: Prostate-specific antigen
BND: Breakend
CNV: Copy number variation
mCRPC: Metastatic castration-resistant prostate cancer
