# Supplementary material for: Genome-wide interrogation of structural variation reveals novel African-specific prostate cancer oncogenic drivers
Source: Genome Med. 2022 Aug 31;14:100. doi: 10.1186/s13073-022-01096-w (PMC9434886; doi:10.1186/s13073-022-01096-w)
Supplement: Supplementary file 1 — Additional file 1: Figure S1. Concordant SV call generation from Manta and GRIDSS. Figure S2. Summary of SVs in each type, compared to other studies. Figure S3. CIRCOS plot of hyper-SV mutated tumours. Figure S4. The spread of SV breakpoints and samples in each 1 Mbp genomic bin. Figure S5. TMPRSS2-ERG fusion with interstitial region retention. Table S1. Clinical and pathological characteristics of 180 prostate cancer patients included in this study. Table S2. Biallelic assessment of CDK12 in hyper-duplicated samples. Table S3. Biallelic assessment of BRCA2 in hyper-deleted samples. [file 13073_2022_1096_MOESM1_ESM.pdf]

Genome-wide Interrogation of Structural Variation Reveals Novel African-specific Prostate Cancer Oncogenic Drivers

Tingting Gong, Weerachai Jaratlerdsiri, Jue Jiang, Cali Willet, Tracy Chew, Sean M. Patrick, Ruth J. Lyons, Anne-Maree Haynes, Gabriela Pasqualim, Ilma Simoni Brum, Phillip D. Stricker, Shingai B.A. Mutambirwa, Rosemarie Sadsad, Anthony T. Papenfuss, Riana M.S. Bornman, Eva K.F. Chan, Vanessa M. Hayes

Supplementary Figures

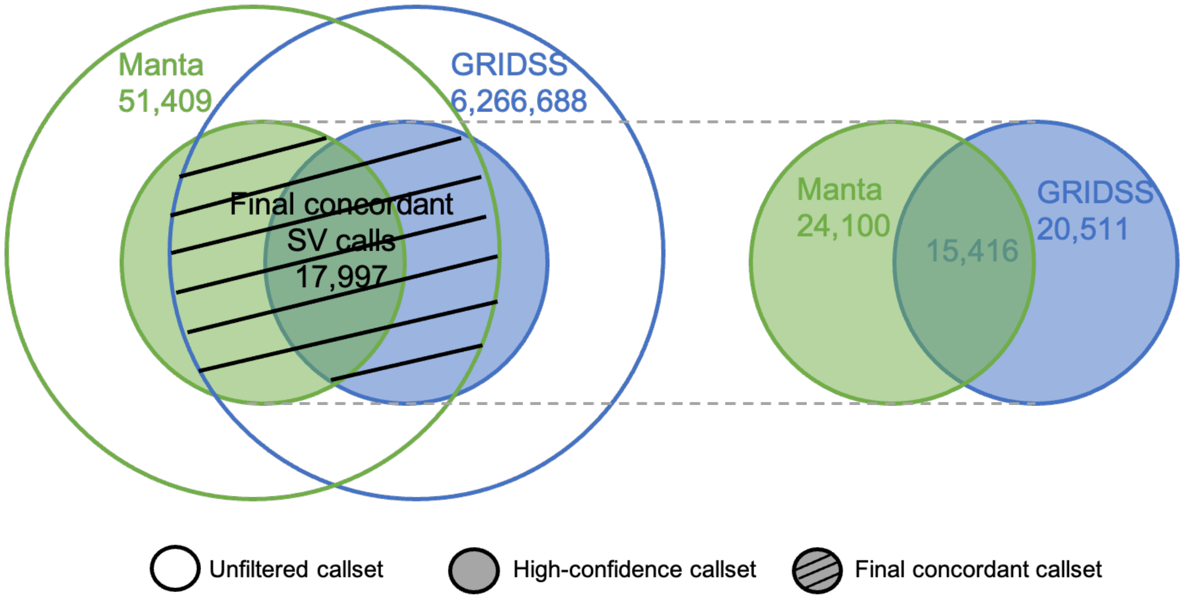

Figure S1. Concordant Structural Variant (SV) call generation from Manta and GRIDSS. Circles represent SV callsets from Manta (green) and GRIDSS (blue). Colour-filled circles represent high-confidence callsets reported. The region in shadow represents SV callsets requiring high-confidence reported by only one of the two callers. Two SV calls were considered as concordant if they have matching SV type and reported breakpoint positions within 5bp of each other. The total number of SV calls found in this cohort were shown for Manta and GRIDSS callset, their high-confidence callset and final concordant callset.

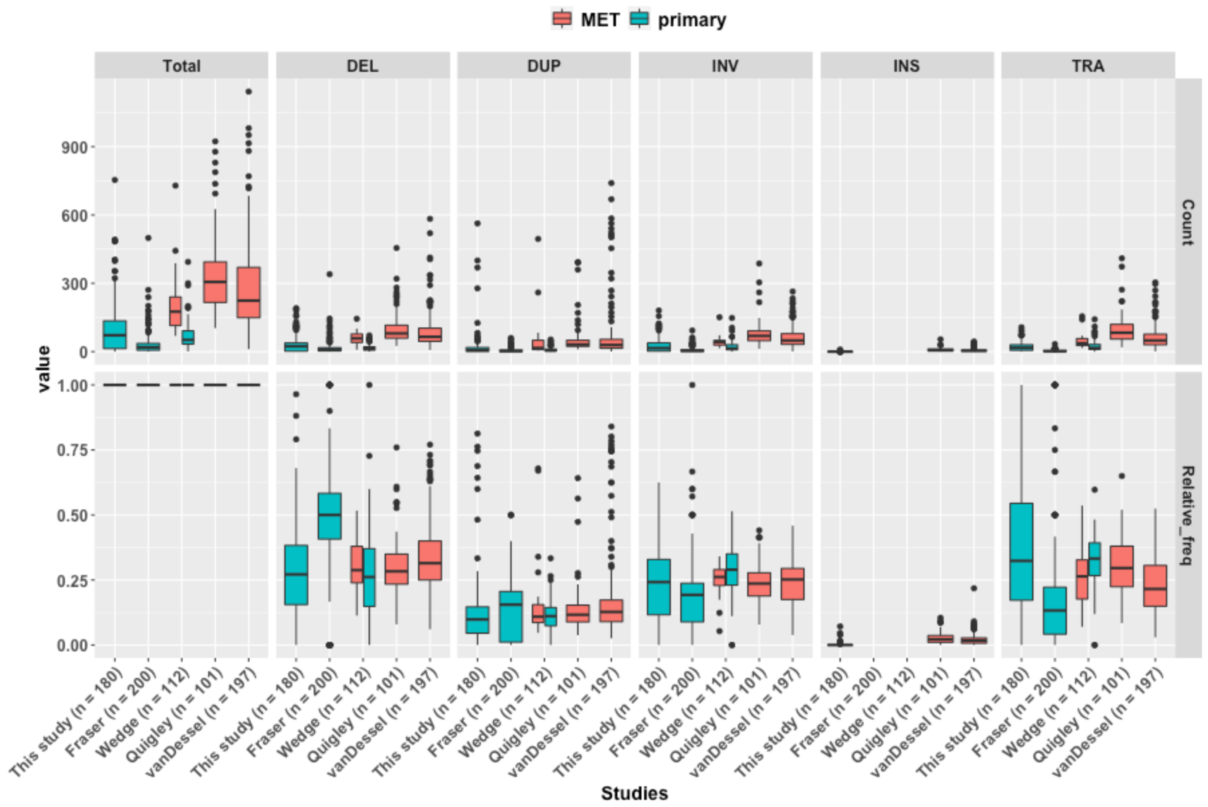

Figure S2. **Summary of Structural Variants (SVs) in each type, compared to other studies.** The top panel represents the spectrum of total count and the bottom one shows the relative frequency of each SV type. Abbreviations: MET, metastatic prostate cancer; primary, primary prostate cancer; DEL, deletion; DUP, duplication; INV, inversion; INS, insertion; TRA, translocation.

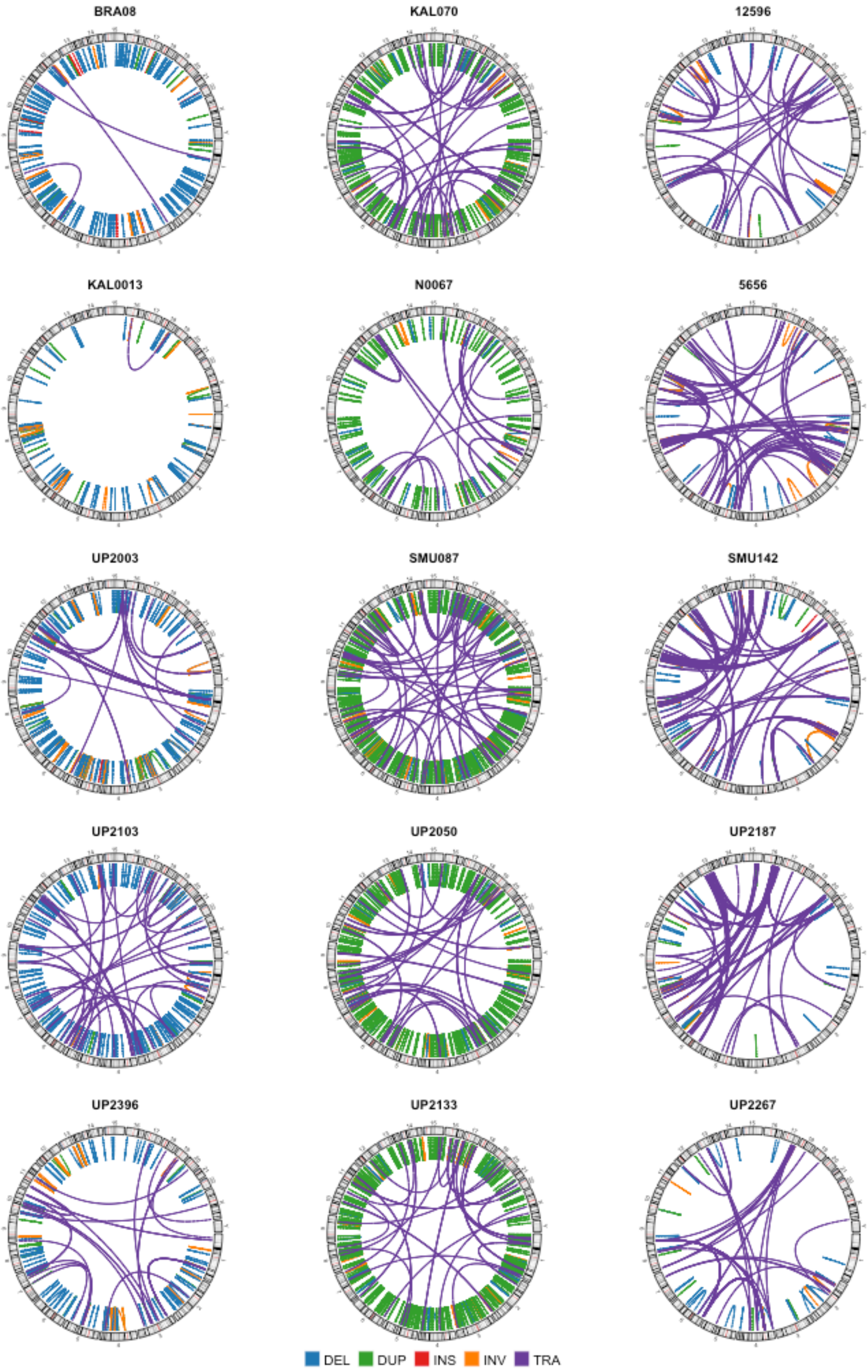

Figure S3. **CIRCOS plot of hyper-SV mutated tumours.** Hyper-deleted, hyper-duplicated and hyper-translocated tumours are shown in left, middle and right column respectively. The sample ID of each tumour is shown on top of each CIRCOS plot.

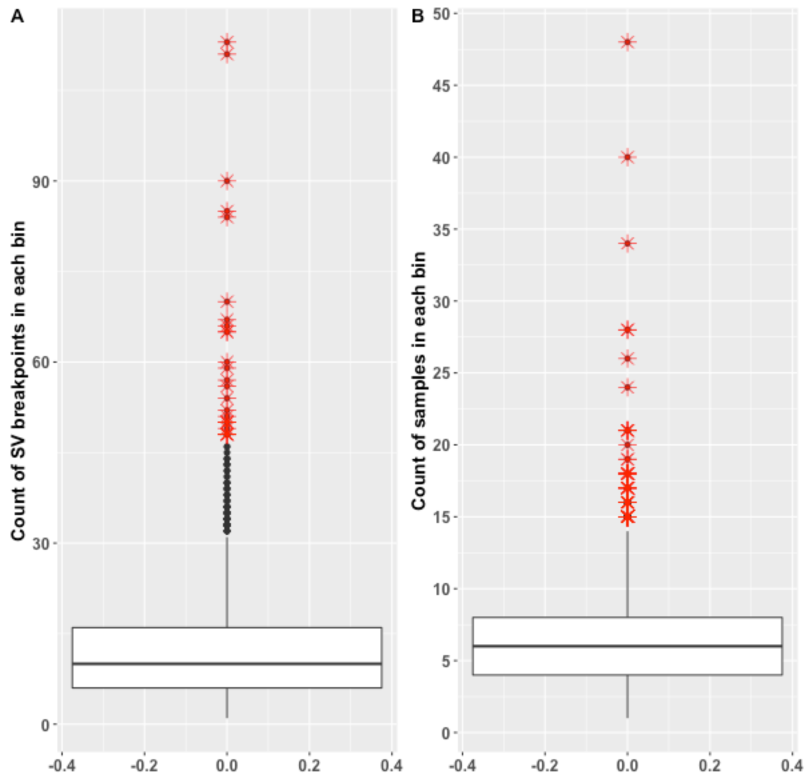

Figure S4. The spread of Structural Variant (SV) breakpoints and samples in each 1 Mbp genomic bin. The outliers highlighted in red are defined as (A) the count of SV breakpoints in each bin > $Q_3 + 3 \times IQR$ and (B) the count of samples in each bin > $Q_3 + 1.5 \times IQR$. $IQR = Q_3 - Q_1$, where Q_1 and Q_3 are first and third quartile respectively.

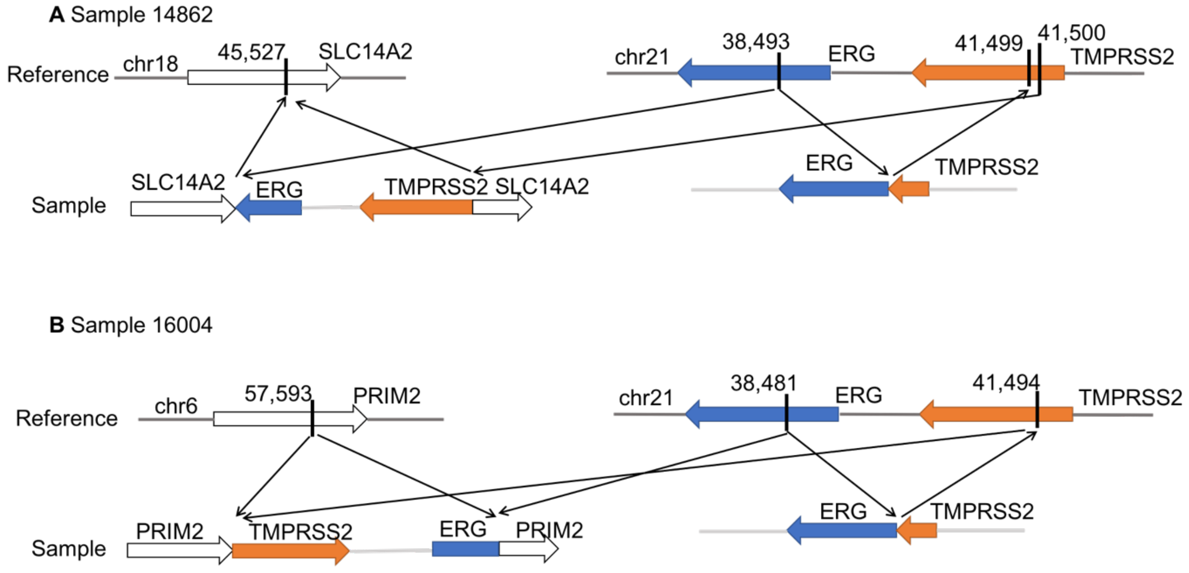

Figure S5. *TMPRSS2-ERG* fusion with interstitial region retention. (A): *TMPRSS2-ERG* fusion with interstitial gene region between *ERG* and *TMPRSS2* translocated to chr18 in sample 14862. (B): *TMPRSS2-ERG* fusion with inverted interstitial gene region between *ERG* and *TMPRSS2* translocated to chr6 in sample 16004. The genomic position indicated in Mbp.

Supplementary Tables

Table S1. Clinical and pathological characteristics of 180 prostate cancer patients included in this study

Variables		Prostate cancer patients		
Ethnic group		African	European	Admixed
Number of patients		115	61	4
Country of origin	South Africa	114	4	2
	Australia	0	53	0
	Brazil	1	4	2
Mean age		67 (45-99, Std dev = 8.4)[2]	62 (46-72, Std dev = 6.0)	68 (63-71, Std dev = 3.8)
Median preoperative PSA[1]		51 (4.3 – 4847)[3]	8.4 (3.5 – 232.2)	16.3 (10 – 2000)
Gleason score status	High-risk (≥ 8)	81	53	4
	Intermediate-risk (= 7)	23	0	0
	Low-risk (= 6)	8	7	0
	unknown	3	1	0

[1]PSA: Prostate Specific Antigen.

[2]One patient in African ancestry has no record of age.

[3]Seven patients in African ancestry have no record of exact PSA.

Table S2. Biallelic assessment of *CDK12* in hyper-duplicated samples

Sample	Ethnicity	Genetic inactivation types	
		**"Loss"**	**"Mutation"**
KAL070	African	• Somatic copy-number loss (CN = -1.293) at 17q12<br>• Somatic DEL at chr17: 39,175,010 - 40,183,454<br>• Somatic DEL at chr17: 39,497,147 - 40,172,259	• Missense germline SNV at chr17:39,526,122 T-A<br>• Missense germline SNV at chr17:39,526,122 T-G
N0067	African	No somatic and germline DEL/loss found	• Missense germline SNV at chr17:39,526,140 C-T<br>• Nonsense somatic SNV (stopgain) at chr17: 39,462,797 T-A<br>• Missense somatic SNV at chr17:39,501,406 A-G
SMU087	African	• Somatic copy-number loss (CN = -0.878) at 17q12<br>• Somatic DEL at chr17: 39,199,568 – 41,440,077	No nonsynonymous somatic or germline SNV
UP2050	African	• Germline DEL at chr17: 36,229,353-62,226,317	• Missense germline SNV at chr17:39,526,122 T-A<br>• Missense germline SNV at chr17:39,526,122 T-G<br>• Missense somatic SNV at chr17-39,509,724-G-T
UP2133	African	• Frameshift deletion at chr17: 39,511,557-TTG-T	• Missense somatic SNV at chr17-39,509,731-G-T

Table S3. Biallelic assessment of *BRCA2* in hyper-deleted samples

Sample ID	Ethnicity	Genetic inactivation types		
		**"Loss"**	**"Break"**	**"Mutation"**
BRA08	European	No somatic and germline DEL/loss	• No somatic/germline breakpoint	• Missense germline SNV at chr13:32332592 A-C<br>• Missense germline SNV at chr13:32355250 T-C
KAL0013	African	No somatic and germline DEL/loss	• No somatic/germline breakpoint	• Missense germline SNV at chr13:32338857 A-G<br>• Missense germline SNV at chr13:32355250 T-C<br>• Missense germline SNV at chr13:32398747 A-G
UP2003	African	• Somatic DEL at chr13: 32,365,812 -36,998,010	• Somatic DEL at chr13:32,315,662 – 32,316,878 (one breakpoint interrupting exon 1: 32,315,479-32,315,667)	• Missense germline SNV at chr13:32332592 A-C<br>• Missense germline SNV at chr13:32355250 T-C<br>• Missense germline SNV at chr13:32379392 A-T
UP2103	African	• Somatic DEL at chr13: 32,377,173 – 35,889,395	• No somatic/germline breakpoint	• Missense germline SNV at chr13:32319100 T-C<br>• Missense germline SNV at chr13:32355250 T-C
UP2396	African	• Somatic DEL at chr13: 31,428,492 – 55,545,422<br>• Frameshift deletion at chr13: 32,340,566-AG-G	• No somatic/germline breakpoint	• Missense germline SNV at chr13:32326142 A-G<br>• Missense germline SNV at chr13:32355250 T-C
